# Prevalence and Associated Predictors of Hypertension in Adult Patients with Thyroid Nodules at the Royal Commission Hospital, Kingdom of Saudi Arabia

**DOI:** 10.26502/fccm.9220303

**Published:** 2023-01-23

**Authors:** Ahmed M Khair, Mona A Sid Ahmed, Faisal H Alharbi, Soha hassan, Nusaiba M Elbadwi, Sami Naji Almutairi, Imad R Musa

**Affiliations:** 1 Royal Commission Hospital at AL Jubail Industrial City, Al Jubail, Kingdom of Saudi Arabia

**Keywords:** Hypertension, Predictors, Prevalence, Thyroid Nodules

## Abstract

**Background:**

Hypertension and thyroid nodules (TNs) are common medical problems that are increasing in prevalence globally. Hence, we conducted this study to assess the prevalence and associated predictors of hypertension in adult patients with TNs at the Royal Commission Hospital, Kingdom of Saudi Arabia (KSA).

**Methods:**

A retrospective study was conducted between 1 January 2015 and 31 December 2021. Patients with documented TNs based on the Thyroid Imaging Reporting and Data System (TI-RADS) were recruited to assess the prevalence and associated risk factors for hypertension.

**Result:**

Three hundred ninety-one patients with TNs were recruited for this study. The median (interquartile range, IQR) age was 46.00 (20.0) years, and 332 (84.9%) of the patients were females. The median (IQR) body mass index (BMI) was 30.26 (7.71) kg/m^2^. There was a high prevalence of hypertension (22.5%) in adult patients with TNs. In the univariate analysis, there were significant associations between diagnosed hypertension in patients with TNs and age, sex, diabetes mellitus (DM), bronchial asthma, triiodothyronine (FT3), total cholesterol and high-density lipoprotein (HDL). In the multivariate analysis, age (OR = 1.076 [95% CI 1.048 – 1.105]), sex (OR = 2.28 [95% CI 1.132 – 4.591]), DM (OR = 0.316 [95% CI 0.175 – 0.573]) and total cholesterol levels (OR = 0.820 [95% CI 0.694 – 0.969]) were significantly associated with hypertension.

**Conclusion:**

There is a high prevalence of hypertension in patients with TNs. Age, female sex, DM and elevated total cholesterol are significant predictors of hypertension in adult patients with TNs.

## 1. Introduction

Thyroid nodules (TNs) are a common clinical problem detected in approximately 5% to 7% of the adult population through physical examination alone. A very high prevalence (50%) of TNs larger than 1 cm in patients without previously diagnosed thyroid disease has been documented in autopsy data [1,2]. Although more than 90% of the detected nodules are clinically insignificant benign lesions [2], TNs are clinically important as they represent thyroid cancer in approximately 4.0% to 6.5% of cases [3]. Moreover, thyroid cancer has emerged as the most common endocrine cancer worldwide, with an incidence of 56 cases per 100,000 [4]. It is also the fifth most common cancer in women and the second most common among those older than 50 years [5]. Currently, TNs are diagnosed more frequently with the use of modern imaging modalities, such as ultrasound, computed tomography (CT), magnetic resonance imaging and positron emission tomography [6]. Likewise, the fine needle aspiration report and scoring systems of thyroid ultrasound are essential tools for evaluating TNs with reliable diagnostic accuracy and prognostic importance [7]. One of the most important risk factors is exposure to ionizing radiation in some environments, along with female gender, age, diabetes mellitus (DM), hypertension and genetics [8–11]. Hypertension is the leading preventable risk factor for cardiovascular disease and all-cause mortality worldwide [12]. A higher prevalence of hypertension among adults has been reported in low- and middle-income countries (31.5%) compared with high-income countries (28.5%) [13]. From 2000 to 2010, the prevalence of hypertension increased by 7.7% in low- and middle-income countries, while it decreased by 2.6% in high-income countries [14]. The associations between TNs and hypertension have been reported in different countries [15–18]. Recently published data from the Kingdom of Saudi Arabia (KSA) have shown higher prevalence of hypertension and prehypertension in the Saudi population [19–21]. In addition, ahigher prevalence has been observed in males compared with females and in the urban compared with the rural population [20,21]. According to the Saudi Heart Association, the economic cost of managing hypertension and its related cardiovascular complications in the KSA is a rapidly growing financial burden that increased from $3.5 billion in 2016 and will likely reach $9.8 billion by 2035 [22]. Meanwhile, thyroid gland disorders are the most common endocrine abnormalities in the KSA and the Middle East region [23,24]. Moreover, a high prevalence of TNs has been reported in the Saudi population (34%–40%) [25,26], along with an increased rate of thyroid cancer (11.7%) [27]. Given the importance of the two abovementioned clinical entities, the potential coexistence of both conditions and the dearth of published data on the topic in the region, the current study aimed to investigate the prevalence of hypertension in adult patients with TNs at the Royal Commission Hospital in the eastern region of the KSA.

## 2. Methods

A retrospective study was conducted at the Royal Commission Hospital from 1 January 2015 to 31 December 2021. The files of adult patients (men and women) aged 18 years and older with documented TNs based on the findings of an ultrasound procedure were retrieved. The ultrasound procedures were conducted in the radiology department of the hospital. Each thyroid ultrasound procedure was performed in the radiology department by a radiology specialist, and each report was reviewed and approved by a radiology consultant. Thyroid ultrasound reports based on the American College of Radiology Thyroid Imaging Reporting and Data System (TI- RADS) were adopted to assess the TNs (Table 1) [28]. Medical records with incomplete data and thyroid ultrasound reports for thyroid ultrasounds conducted in other hospitals were excluded. Finally, 391 patients with TNs were recruited for this study. The socio-demographic data, including each patient’s age, sex, weight, height, thyroid status and common co-morbidities (type 1 DM and type 2 DM, hypertension and bronchial asthma) were gathered using a data collection sheet. Moreover, laboratory tests for haematological indices (white blood cell count, haemoglobin and platelet count), thyroid function, lipid profiles and vitamin D levels were collected.

### 2.1 Definition of Variables

#### 2.1.1 Thyroid Nodules:

TNs were diagnosed according to the ACR TI-RADS classification criteria for risk assessment of malignant TNs [28].

#### 2.1.2 Body Mass Index (BMI):

BMI was calculated as the body mass divided by the square of the body height; it is expressed in units of kg/m², resulting from mass in kilograms and height in metres.

#### 2.1.3 Hypertension:

This variable included patients known to be hypertensive and receiving treatment according to their medical records during the time the thyroid ultrasound was conducted.

#### 2.1.4 DM:

This variable includes those who had documentation of DM (type 1 and 2), whether they were on diet control or glucose-lowering drugs during the time of the thyroid ultrasound procedure.

#### 2.1.5 Bronchial Asthma:

This variable included patients diagnosed with bronchial asthma based on their medical records around the time of the thyroid ultrasound procedure.

#### 2.1.6 Vitamin D Deficiency:

This variable was defined as a 25-hydroxyvitamin D (25[OH]D) level of < 30 ng/mL; levels equal to or above this cut-off point were considered normal [29].

### 2.2 Statistical Analysis

Data were analysed using SPSS for Windows (version 22.0). The Shapiro-Wilk test assessed the normality for continuous data; all variables were not normally distributed. Data were expressed as proportions, medians with interquartile ranges (IQRs) or numbers and proportions, as applicable. A univariate analysis was performed, with the diagnosed hypertension as the dependent variable. The independent variables were age, sex, BMI, thyroid status, DM, bronchial asthma, thyroid hormones, 25(OH)D level, haematological indices (haemoglobin, white blood cell count and platelet count), lipid profile and TI-RADS ultrasound score. A variable was analysed using logistic regression if its univariate P-value was < 0.20, and backward stepwise likelihood ratio regression was selected for adjustment. Odds ratios (ORs) and 95% confidence intervals (CIs) were computed, and P-values < 0.05 were considered significant.

## 3. Results

Three hundred ninety-one patients with documented TNs were recruited for the study. The median (IQR) for the patients’ age was 46.00 (20.0) years, and the majority were females (84.9%). The median (IQR) for the BMI and 25(OH)D level were 30.26 (7.71) kg/m^2^ and 14.50 (12.0) nmol/L, respectively. The median (IQR) values for the thyroid hormones were as follows: thyroid-stimulating hormone (TSH), 1.72 (2.43) mmol/L; free thyroxine, 1.12 (0.45) ng/dL; and FT3, 2.69 (0.40) nmol/L. While most participants had normal thyroid functions (64%), a small percentage of patients had either hypothyroidism (28.6%) or hyperthyroidism (7.4%). The median (IQR) for total cholesterol, low-density lipoprotein (LDL), high-density lipoprotein (HDL) and triglycerides were 5.80 (3.89) mmol/L, 3.76 (0.80) mmol/L, 2.99 (1.71) mmol/L and 1.78 (1.11) mmol/L, respectively. The median (IQR) values for the haematological indices were as follows: white blood cell count, 7.01 (2.63) 10^9^/L; haemoglobin, 12.60 (1.7) gm/dl; and platelet count, 276.15 (95.8) 10^3^/dl. The patients’ percentages according to the TI-RADS ultrasound scores (1,2, 3, 4 and 5) were 2.6%, 18.4%, 40.7%, 36.3% and 2%, respectively (Table 2). There was a high prevalence of hypertension (22.5%) in adult patients with TNs.

In the univariate analysis, there was no association between diagnosed hypertension in adult patients with TNs and BMI, 25(OH)D level, thyroid status, haematological indices (white blood cell count, haemoglobin and platelet), LDH, triglycerides, TSH, FT4 and thyroid ultrasound based on the TI-RADS scoring system. However, there were significant associations between diagnosed hypertension in adult patients with TNs and age (OR = 1.099 [95% CI 1.073 – 1.127]), sex (OR = 3.14 [95% CI 1.750 – 5.634]), DM (OR = 0.160 [95% CI 0.095 – 0.27]), bronchial asthma (OR = 0.495 [95% CI 0.227 – 1.077]), FT3 (OR = 0.666 [95% CI 0.468 – 0.948]), total cholesterol (OR= 0.645 [95% CI 0.559 – 0.745]) and HDL (OR = 0.420 [95% CI 0.316 – 0.558]), Table 3).

In the multivariate analysis, bronchial asthma, FT3 and HDL were not significantly associated with diagnosed hypertension in adult patients with thyroid nodular diseases. However, there were significant associations between hypertension in adult patients with TNs and age (OR = 1.076 [95% CI 1.048 – 1.105], P < 0.000), sex (OR = 2.28 [95% CI 1.132 – 4.591], P = 0.002), DM (OR = 0.316 [95% CI 0.175 – 0.573], P = 0.040) and total cholesterol levels (OR = 0.820 [95% CI 0.694 – 0.969], P = 0.020) (see Table 4).

## 4. Discussion

The current study documented a higher prevalence of hypertension (22.5%) in adults with thyroid nodular diseases in the KSA. The prevalence of hypertension in patients with TNs in this study was higher than that obtained recently in the KSA (16.8%) [18] and slightly lower than that reported in Turkey (26.9%) [17]. Surprisingly, a very high prevalence of hypertension in a similar group of patients was demonstrated in China (45.6%) [30], particularly in the Daxing District of Beijing (60.37%) [31] and those with advanced age (69.8%) (mean age =102.8±2.8 years) [32]. It is worth mentioning that a higher prevalence of untreated hypertension (62.7%) compared with treated hypertension (56.1%) was documented in the Chinese population with TNs in Beijing, China [31]. The significant association between hypertension and the presence of TNs has been reported in many studies in different countries across the globe, such as the KSA [18], Turkey [15,17], China [11,16,30–33], Korea 34 and Japan [35,36]. The higher prevalence of hypertension obtained in patients with TNs may be attributed to the higher prevalence of hypertension reported recently in the KSA (37.5%) [19]. Likewise, a report in 2007 from the KSA showed a higher prevalence of hypertension in males (28.6%) versus females (23.9%) and in rural (22.4%) versus urban (27.9%) population [21]. Additionally, a higher prevalence of prehypertension was documented recently among Saudi men (47.2%) and women (24.7%) [20]. The high prevalence of hypertension in our study was in concordance with the global rise in the prevalence of hypertension in low- and middle-income countries (31.5%) and with the higher prevalence of hypertension in high- income countries (28.5%), despite being decreased by 2.6% [13]. Interestingly, a family history of hypertension was noted in 26.9% of patients with TNs [32]. G-protein-coupled receptor kinases (GRKs) are implicated in the pathophysiology of arterial hypertension and thyrotropin receptor (TSHR). Their role in the pathophysiology of hyperfunctioning TNs can be promoted as the explanation for the association of hypertension and thyroid nodular diseases [37]. Another extrapolation regarding this association is that the high level of aldosterone concentration and the presence of aldosterone synthase expression suggest that aldosterone may be locally produced and secreted in thyroid tissues from cystic nodules [38]. Similarly, recently published data from Japan underscored a significant positive association between thyroid cysts and hypertension [35] and systolic hypertension [36], indicating a higher thyroid hormone activity in thyroid cysts [35]. The current study showed older age as a significant predictor of the presence of hypertension in adult patients with TNs. Similar significant associations of age and hypertension in the same group of participants have been documented recently in many published studies [15,16,30,31,33,34], in line with the results of previous research revealing that the higher prevalence of hypertension and TNS are associated with increased age [15,32,34,39]. This may be linked to inflammation, the most consistently documented biological feature of aging, which entails higher inflammatory markers [39]. Moreover, the higher prevalence of hypertension and TNs have been associated with the prevalence of a wide range of age-related co-morbidities, such as cardiovascular co-morbidities [40], insulin resistance and DM [41]. On the other hand, the degeneration of thyroid cells reflects the age-related changes in the thyroid gland, leading to fibrosis, infiltration of inflammatory cells, thyroid follicle alteration and thyroid nodular diseases [42]. Our study and other studies worldwide have shown the higher prevalence of TNs among females and demonstrated that the female sex is a significant predictor of hypertension in adult patients with TNs [15,16,30,34]. This may be explained by the effects of oestrogen that promotes the growth of thyroid stem cells and progenitor cells, leading to the proliferation of thyroid stem cells and TN formation through different mechanisms (e.g. classical genomic and non-genomic pathways) [43,44]. A study in 2018 from the United States demonstrated an increase in hypertension prevalence and associated cardiovascular complications among females [45]. Moreover, the gender difference has been well established in the prevalence of hypertension: women accounted for approximately 51% of the hypertensive population based on the Seventh/Eighth Report of the Joint National Committee on Prevention, Detection, Evaluation, and Treatment of High Blood Pressure (JNC 7/8) Guidelines using the National Health and Nutrition Examination Survey (NHANES) blood pressure data from 2011–2014 [45]. Likewise, after menopause, women display a more rapid increase in the prevalence of hypertension compared with men, eventually exceeding that seen in men at age 75 and above [45]. In this study, the presence of DM is a highly significant predictor of hypertension in adult patients with thyroid nodular diseases. This was strengthened by the results of many clinical trials documenting that both DM and hypertension are positively and significantly associated with TNs [10,11,15,17,30,31,33,34]. Moreover, the risk of getting any of the three co- morbidities (hypertension, DM and TNs) increases with aging [32,40,41]. Furthermore, aging promotes TN formation with the risk of associated metabolic syndrome [10,30,31], insulin resistance and high levels of insulin-like growth factor-1 [11,41]. Our study and many other studies have found that a higher level of total cholesterol is a significant predictor of the presence of hypertension in adult patients with TNs [10,16,31,34,46], indicating the positive and significant association of the components of metabolic syndrome and the formation of TNs associated with insulin resistance, insulin-like growth factor-1 and high level of inflammatory markers [10,11,46]. Moreover, a high level of total cholesterol can predict the risk of thyroid cancer [47]. Recently, some studies have indicated that cholesterol influences the signal pathway of growth factor receptors in tumour cells, inducing an abnormal pathological signal and causing carcinogenesis [47]. Likewise, cholesterol may potentiate increased tumour angiogenesis and proliferation and reduced apoptosis of tumour cells [48]. The results of the present study are in accordance with the outcomes of many studies that have documented the lack of significant associations between TNs and other associated predictors (thyroid hormones, thyroid status, haematological indices, vitamin D levels and the TI-RADS scoring system) [11,49,50].

## 5. Limitation

This study was retrospective and involved only one centre. Other factors, such as thyroid antibodies, iodine levels, blood pressure control, duration of hypertension, nutritional patterns, smoking, alcohol consumption, exposure to radiation, genetic analysis and environmental factors, were not assessed.

## 6. Conclusion

There is a high prevalence of hypertension in adult patients with thyroid nodular disease. Age, female sex, DM and a high level of total cholesterol are significant predictors of hypertension in patients with TNs in the eastern region of the KSA.

## Figures and Tables

**Table 1:** (TI-RADS) Category definitions.

TI-RADS -1	Benign
TI-RADS -2	Not suspicion
TI-RADS -3	Mildly suspicion
TI-RADS -4	Moderately suspicion
TI-RADS -5	Highly suspicion

TI-RADS, thyroid imaging reporting and data system.

**Table 2:** general characteristics of adult patients with thyroid nodules in eastern region 2015-2021.

Variables		Median	Interquartile range
Age, years		46	20
Body mass index, kg/m^2^		30.26	7.71
Vitamin d , nmol/L		14.5	12
Thyroid-stimulating hormone, mmol/L		1.71	2.43
Free triiodothyronine, nmol/L		2.69	0.4
Free thyroxine, ng/dL		1.12	0.45
Haemoglobin, gm/dl		12.6	1.7
White blood cell, 10^9^/L		7.01	2.63
Platelet, 10^3^/dl		276.15	95.8
Total cholesterol, mmol/L		5.8	3.89
Low-density lipoprotein, mmol/L		3.76	0.8
High-density lipoprotein, mmol/L		2.99	1.71
Triglyceride, mmol/L		1.78	1.11
		Number	Proportion
Sex	Female	332	84.9
	Male	59	15.1
Diabetes mellitus	No	297	76
	Yes	94	24
Hypertension	No	303	77.5
	Yes	88	22.5
Asthma	No	360	92.1
	Yes	31	7.9
Thyroid status	Euthyroid	250	64
	Hypothyroidism	112	28.6
Ultrasound based on TI-RADS	Hyperthyroidism	29	7.4
	TI-RADS 1	10	2.6
	TI-RADS 2	72	18.4
	TI-RADS 3	159	40.7
	TI-RADS 4	142	36.3
	TI-RADS 5	8	2

**Table 3:** Univariate analysis of the factors associated with hypertension in adult patients with thyroid nodules in eastern region 2015- 2021.

Variables		hypertension (n=88)	No hypertension (n=303)	OR (95.0 %CI	P
		Median			
Age, years		57.00 (13.8)	43.00 (19.0)	1.099 (1.073 – 1.127)	<0.000
Body mass index, kg/m^2^		32.73(7.57)	29.48 (7.32)	1.004 0(.995 – 1.013)	0.407
Vitamin D		17.1 (13.93)	13.9 (11.43)	1.001(0.995 – 1.008)	0.707
Thyroid-stimulating hormone, mmol/L		1.64 (2.55)	1.71 (2.41)	1.005 (0.995 – 1.016)	0.315
Free triiodothyronine, nmol/L		2.691(0.50)	2.69 (.38)	0.666 (0.468 – 0.948)	0.024
Free thyroxine, ng/Dl		1.19(0.40)	1.10 (0.51)	0.894 (0.729 – 1.098)	0.285
haemoglobin, gm/dl		12.70(1.86)	12.50 (1.80)	0.971(0.886–1.064)	0.524
White blood cell, 10^9^/L		7.40 (2.65)	6.90 (2.79)	0.997 (0.982 – 1.012)	0.701
Platelet, 10^3^/dl		275.58(99.5)	276.2 (85.7)	0.0998 (0.996 – 1.001)	0.201
Total cholesterol, mmol/L		4.79(1.90)	6.31(3.68)	0.645(0.559 – 0.745)	<0.000
Low-density lipoprotein, mmol/L		2.94(1.60)	3.76 (0.47)	1.000 (0.950 – 1.053)	0.995
High-density lipoprotein, mmol/L		1.32 (1.90)	3.00 (1.47)	0.420(0.316 – 0.558)	0
Triglyceride, mmol/L		1.48(01.15)	1.97 (1.08)	0.824(0.587 – 1.156)	0.263
Gender	Male	25 (28.4)	34 (11.2)	Reference	<0.000
	Female	63 (71.6)	269 (88.8)	3.140 (1.750 – 5.634)	
Thyroid status	Euthyroid	54 (61.4)	196 (64.7)	Reference	
	Hypothyroidism	27 (30.7)	85 (28.1)	0.866 (0.351 – 2.135)	0.754
	Hyperthyroidism	7 (8.0)	22 (7.3)	0.998 (0.384 – 2.593)	0.997
Diabetes mellitus	No	41(46.6)	256 (84.5)	Reference	<0.000
	Yes	47 (53.4)	47 (15.5)	0.16 (0.095 – 0.27)	
Bronchial asthma	No	77 (87.5)	283 (93.4)	Reference	0.076
	Yes	11 (12.5)	20 (6.6)	0.495 (0.227 – 1.077)	
Ultrasound	TIRADS1	2 (2.3)	8 (2.6%)	Reference	
	TIRADS2	14 (15.9)	58 (19.1)	1.75 (0.129 – 23.703)	0.674
	TIRADS3	36 (40.9)	123 (40.6)	1.69 (0.192 – 14.873)	0.636
	TIRADS4	35 (39.8)	107 (35.3)	2.049 (0.244 – 17.205)	0.509
	TIRADS5	1 (1.1)	7 (2.3)	2.290 (0.272 – 19.263)	0.446

IQR- Interquartile Range; OR- Odds Ratio; CI- Confidence Interval.

**Table 4:** Multivariate analysis of the factors associated with hypertension in adult patients with thyroid nodules eastern region 2015- 2021.

Variables		OR (95.0 %CI	P
Age, years		1.076 (1.048 – 1.105)	<0.000
Free triiodothyronine, nmol/L		0.700 (0.444 – 1.104)	0.125
Total cholesterol		0.820 (0.694 – 0.969)	0.020
High-density lipoprotein, mmol/L		0.780 (0.549 – 1.108)	0.165
Diabetes mellitus	No	Reference	
	Yes	0.316 (0.175 – 0.573)	0.040
Bronchial asthma	No	Reference	
	Yes	0.696 (0.269 – 1.798)	0.454
Sex	Male	Reference	
	Female	2.28 (1.132 – 4.591)	0.002

IQR- Interquartile Range; OR- Odds Ratio; CI- Confidence Interval

## Data Availability

Upon request, please contact the corresponding author (I R.Musa).

## Abbreviations:

KSA: Kingdom of Saudi Arabia
DM: Diabetes Mellitus
T2DM: Type 2 Diabetes Mellitus
T1DM: Type 2 Diabetes Mellitus
TNs: Thyroid Nodules
FT4: Free Thyroxine
FT3: Free Triiodothyronine
T3: Hormone Triiodothyronine
T4: Thyroxine
TSH: Thyroid-Stimulating Hormone
25(OH)D: 25-hydroxyvitamin D
LDL: Low-Density Lipoprotein
HDL: High-Density Lipoprotein
AOR: Adjusted Odds Ratio
IQR: Interquartile Range
kg: kilogram
m: Meter
cm: Centimeter
BMI: Body Mass Index
CI: Confidence Interval
SD: Standard Deviation
ACR TI- RADS: American College of Radiology Thyroid Imaging Reporting and Data System
